# The American Institute at Deer Park

**Published:** 1884-04

**Authors:** 


### THE AMERICAN INSTITUTE AT DEER PARK.

We have received a very polite invitation from the B. & O. R. R. Co. to accept the courtesy of their road should we attend the coming meeting of the Institute at Deer Park. We are grateful, but must decline. The annual meetings of the "junketing association" are not in our line.

The B. & O. Co. have a fine railroad and an elegant hotel at Deer Park; now this much being supplied by the B. & O. Co., could they not finish the job by furnishing a large supply of good Homœopathy? *then* the meeting would be worth attending.

